# Interactions between PPAR Gamma and the Canonical Wnt/Beta-Catenin Pathway in Type 2 Diabetes and Colon Cancer

**DOI:** 10.1155/2017/5879090

**Published:** 2017-02-19

**Authors:** Yves Lecarpentier, Victor Claes, Alexandre Vallée, Jean-Louis Hébert

**Affiliations:** ^1^Centre de Recherche Clinique, Hôpital de Meaux, Meaux, France; ^2^Department of Pharmaceutical Sciences, University of Antwerp, Wilrijk, Belgium; ^3^CHU Amiens Picardie, Université Picardie Jules Verne, Amiens, France; ^4^Experimental and Clinical Neurosciences Laboratory, INSERM U1084, University of Poitiers, France; ^5^Institut de Cardiologie, Hôpital de la Pitié-Salpêtrière, Assistance Publique-Hôpitaux de Paris, Paris, France

## Abstract

In both colon cancer and type 2 diabetes, metabolic changes induced by upregulation of the Wnt/beta-catenin signaling and downregulation of peroxisome proliferator-activated receptor gamma (PPAR gamma) may help account for the frequent association of these two diseases. In both diseases, PPAR gamma is downregulated while the canonical Wnt/beta-catenin pathway is upregulated. In colon cancer, upregulation of the canonical Wnt system induces activation of pyruvate dehydrogenase kinase and deactivation of the pyruvate dehydrogenase complex. As a result, a large part of cytosolic pyruvate is converted into lactate through activation of lactate dehydrogenase. Lactate is extruded out of the cell by means of activation of monocarboxylate lactate transporter-1. This phenomenon is called Warburg effect. PPAR gamma agonists induce beta-catenin inhibition, while inhibition of the canonical Wnt/beta-catenin pathway activates PPAR gamma.

## 1. Introduction

In numerous mammalian living cells, PPAR gamma and the canonical Wnt/beta-catenin pathway behave in an opposite manner [1–5]. Beta-catenin and PPAR gamma interact with each other in a mechanism that alters each of their activities [6]. In several diseases, PPAR gamma is upregulated while canonical Wnt/beta-catenin is downregulated [7] such as in arrhythmogenic right ventricular cardiomyopathy (ARVC), osteoporosis, and certain neurodegenerative diseases (Alzheimer's disease [8], bipolar disorder, and schizophrenia). Conversely, in other diseases, PPAR gamma is downregulated while canonical Wnt/beta-catenin is upregulated such as in type 2 diabetes, cancers, and certain neurodegenerative diseases (amyotrophic lateral sclerosis [9], Parkinson's disease, Huntington's disease, multiple sclerosis, and Friedreich's ataxia). PPAR gamma agonists induce beta-catenin inhibition in several cellular systems [1, 3, 4, 10]. Moreover, inhibition of canonical Wnt/beta-catenin pathway induces activation of PPAR gamma [11–13]. Nonsteroidal anti-inflammatory drug inhibition of beta-catenin in malignant cells requires a high level expression of PPAR gamma and its coreceptor retinoid-X-receptor alpha [14]. In terms of PPAR gamma and Wnt/beta-catenin signaling, both type 2 diabetes and colon cancer share several similarities from a metabolic point of view. In the two diseases, upregulation of the canonical Wnt system leads to activation of pyruvate dehydrogenase kinase (PDK), which decreases the activity of the pyruvate dehydrogenase complex (PDH). Thus, pyruvate cannot be totally converted into acetyl-coenzyme which does not the mitochondrial TCA cycle. Conversely, PPAR gamma activation selectively decreased PDK mRNA [15]. The multiple and complex properties of these two major pathways, particularly in glucose regulation and cell proliferation, may partly account for the association frequently observed between type 2 diabetes and colon cancer.

## 2. Link between Type 2 Diabetes and Colon Cancer

The association between type 2 diabetes and cancer, including pancreatic and endometrial carcinoma, breast cancer, and colorectal and bladder cancers, has been known for many years. Epidemiological studies have reported a link between type 2 diabetes, obesity, and cancer, especially colon cancer [16–19]. Type 2 diabetes associated with obesity represents a major risk factor for cancer [20–24]. Shared risk factors for colorectal cancer and type 2 diabetes include obesity, physical inactivity, and ageing. Patients with type 2 diabetes present a 30–40% higher risk of developing colon cancer compared to those without diabetes. Type 2 diabetes risk variants also contribute to the risk of colorectal cancer [25]. Metformin, an antidiabetic agent, decreases cancer mortality in diabetic patients [26].

## 3. Underlying Molecular Basis for the Link between Diabetes and Colon Cancer

The underlying molecular basis for the link between type 2 diabetes and colon cancer is not fully understood. Hyperinsulinemia provides a link between diabetes, obesity, and cancer. Hyperinsulinemia and/or insulin resistance represent major factors in cancer pathogenesis [27]. The hypothesis for the association between diabetes and cancer is based on the fact that, in type 2 diabetes, hyperinsulinemia promotes the growth of cancer cells [28]. In colon cancer, the hyperinsulinemia hypothesis suggests that elevated levels of both insulin and free insulin growth factor (IGF-1) promote cell proliferation and enhancement of cell transformation, ultimately resulting in colorectal cancer [29]. High insulin levels represent an adaptive process to insulin resistance at the onset of type 2 diabetes. Cancers overexpress receptors for insulin, including insulin receptor A and IGF-1 receptor. Increased insulin/IGF signaling favors the proliferative properties of the two hormones. Moreover, hyperglycemia and chronic inflammation may also play a role in promoting cancer growth [30].

## 4. Activation of Canonical Wnt Signaling Induces Aerobic Glycolysis or Warburg Effect

### 4.1. Canonical Wnt/Beta-Catenin Pathway (Figures 1 and 2)

**Figure 1:**
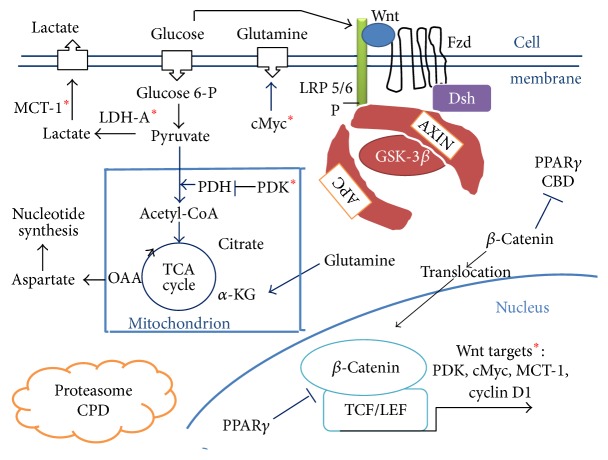
A model of interactions between the canonical Wnt/beta-catenin pathway and PPAR gamma under aerobic glycolysis conditions in colon cancer. In the absence of the Wnt ligand (“off state”), cytosolic beta-catenin is phosphorylated by GSK-3 beta. APS and AXIN combine with GSK-3 beta and beta-catenin to enhance the destruction process in the proteasome (beta-catenin proteasomal degradation: CPD). In the presence of the Wnt ligand (“on state”), Wnt binds both Frizzled and LRP5/6 receptors to initiate LRP phosphorylation and dishevelled-mediated Frizzled internalization. This leads to dissociation of the AXIN/APC/GSK-3 beta complex. Beta-catenin phosphorylation is inhibited. Thus, beta-catenin accumulates in the cytosol and then translocates to the nucleus to bind TCF-LEF cotranscription factors, which induce the Wnt-response gene transcription (PDK, MCT-1, cMyc, and cyclin D1). Glucose itself activates the Wnt pathway. PPAR gamma via APC activates CPD. PPAR gamma inhibits the beta-catenin-TCF/LEF complex. Beta-catenin binds PPAR gamma CBD. PDK inhibits the PDH complex in mitochondria. Thus pyruvate cannot be converted into acetyl-CoA and enters the TCA cycle. Myc activates LDH-A which converts cytosolic pyruvate into lactate. MCT-1 favors lactate secretion from the cytosol which favors angiogenesis. cMyc increases glutamine entry in the cytosol and mitochondria. Myc-induced glutamine enhances nucleotide synthesis. Abbreviations are as follows: adenomatous polyposis coli (APC); alpha ceto-glutarate (a-KG); beta-catenin proteasomal degradation: CPD; catenin binding domain (CBD); Dishevelled (Dsh); Frizzled (Fzd); glycogen synthase kinase-3beta (GSK-3beta); lactate dehydrogenase (LDH); low-density lipoprotein receptor-related protein 5/6 (LRP5/6); monocarboxylate lactate transporter-1 (MCT-1); peroxisome proliferator-activated receptor gamma (PPAR gamma); pyruvate dehydrogenase complex (PDH); pyruvate dehydrogenase kinase (PDK); T-cell factor/lymphoid enhancer factor (TCF/LEF); tricarboxylic acid (TCA); *∗*: Wnt targets: PDK, cMyc, MCT-1, and cyclin D1.

**Figure 2:**
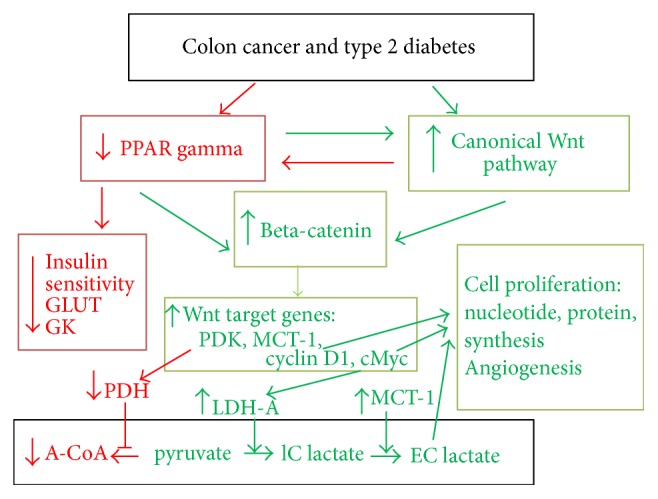
A schematic representation of interactions between PPAR gamma and the canonical Wnt/beta-catenin pathway in type 2 diabetes and colon cancer. Green arrow: activation; red arrow: inhibition; abbreviations are as follows: acetyl-CoA (A-CoA); glucokinase (GK); glucose transporter (GLUT); intracellular lactate (IC lactate); extracellular lactate (EC lactate); lactate dehydrogenase-A (LDH-A); monocarboxylate lactate transporter-1 (MCT-1); pyruvate dehydrogenase (PDH); pyruvate dehydrogenase kinase (PDK); peroxisome proliferator-activated receptor gamma (PPAR gamma).

The Wnt/beta-catenin signaling plays an important role in cell fate, epithelial-mesenchymal transition (EMT) signaling, and embryonic development. Its dysfunction is involved in several pathologies such as carcinogenesis [31–34]. The major effector of the canonical Wnt pathway is the transcription factor beta-catenin/T-cell factor/lymphoid enhancer factor (TCF/LEF). In the absence of Wnt, the free cytosolic beta-catenin is phosphorylated and is tightly controlled by a destruction complex, consisting of AXIN, tumor suppressor adenomatous polyposis coli (APC), and glycogen synthase kinase-3 (GSK-3beta). The destruction complex interacts with beta-catenin and phosphorylates it. The phosphorylated beta-catenin is then degraded in the proteasome (beta-catenin proteasomal degradation: CPD). In the presence of ligands, the Wnt receptor interacts with the Frizzled (Fzd) receptor and LDL receptor-related protein 5/6 (LRP5/6) coreceptors. The Wnt receptor associates with Dishevelled protein (Dsh). This triggers the disruption of the destruction complex and prevents CPD. Beta-catenin then translocates to the nucleus and interacts with TCF/LEF which stimulates the beta-catenin downstream target genes (PDK, MTC-1, cMyc, cyclin D1, Cox 2, AXIN 2, etc.) [35–38] (Figures 1 and 2).

### 4.2. Canonical Wnt Pathway and Glucose

Importantly, glucose itself can directly impact the canonical Wnt pathway [39]. In cancer cells, glucose-induced beta-catenin acetylation favors the Wnt pathway. High glucose level enhances the nuclear translocation of beta-catenin in response to Wnt signaling. Increased glucose consumption is characteristic of cancer cells and high serum glucose levels may modulate cancer-related signaling.

### 4.3. Aerobic Glycolysis in Cancer Cells: The Warburg Effect

The role of the Wnt pathway in driving cell proliferation during oncogenesis and especially colon cancer is well-known [40]. On the one hand, overactivation of canonical Wnt/beta-catenin signaling via TCF/LEF leads to cell proliferation, migration, angiogenesis, and EMT signaling [41–43]. On the other hand, the Wnt pathway induces aerobic glycolysis allowing glucose utilization for cancer cell proliferation [38, 44]. In cancer cells, a large proportion of the glucose supply is fermented in lactate regardless of the availability of oxygen. This phenomenon is called aerobic glycolysis or Warburg effect [45] and ultimately leads to anabolic production of biomass, that is, nucleotide synthesis [46, 47]. As a consequence, in the Warburg effect, a large part of cytosolic pyruvate is not converted into acetyl-CoA which does not enter the TCA cycle. PDK1, a key regulator of glycolysis, phosphorylates the PDH complex which partially inhibits the conversion of pyruvate to acetyl-CoA into mitochondria [48]. PDK1 is upregulated in colon cancer [38]. Thus, cytosolic pyruvate is converted into lactate through activation of LDH-A. Moreover, upregulation of MCT-1 diverts pyruvate towards lactate secretion from the cell. Aerobically derived lactate stimulates angiogenesis [49]. Thus, most of the cytosolic pyruvate is converted into lactate, which is secreted from the cell, and not oxidized in the mitochondrial TCA cycle, despite the availability of oxygen.

In colon cancer, it has recently been shown that activation of the canonical Wnt/beta-catenin pathway partly decreases the oxidative metabolism in the TCA cycle and promotes cell proliferation [38]. Both PDK1 and the lactate transporter MCT-1 are Wnt/beta-catenin targets and are overexpressed in cancer cells. Moreover, the Wnt pathway induces the transcription of genes involved in cell proliferation, that is, cMyc (through glutaminolysis, nucleotide synthesis, and LDH-A activation) and cyclin D1 (through G1) [50–55]. The Wnt target gene cMyc drives aerobic glycolysis and glutaminolysis [52, 54, 56]. Myc also induces LDH-A activation (for conversion of cytosolic pyruvate into lactate). cMyc induces glutamine uptake into the cell and the mitochondria and favors aspartate synthesis [52] (Figures 1 and 2). Through the Warburg effect, cMyc-induced glutaminolysis favors nucleotide synthesis. cMyc also increases the hypoxia-inducible factor-1 alpha- (HIF-1alpha-) mediated control of PDK1 [57].

Thus, in colon cancer, activation of canonical Wnt signaling directly acts on aerobic glycolysis and increases vessel development via the Wnt target gene PDK1 [38]. Part of the pyruvate is converted into acetyl-CoA which enters the TCA cycle and is converted into citrate, which promotes protein synthesis. Cellular accumulation of metabolic intermediates (aspartate, serine, glycine, and ribose) allows de novo nucleotide synthesis, which contributes to growth and proliferation (Figure 1). Moreover, blocking Wnt reduces PDK1 levels via the transcription regulation and reduces in vivo tumor growth. PDK1 is upregulated in several cancers, especially colon cancer [58–60]. Likewise, PDK1 and PDK2 enhance angiogenesis [61, 62]. PDK1 favors vascularization [38]. Angiogenesis is also favored by lactates [63]. MCTs are also upregulated in colon cancer [64].

## 5. Pyruvate Dehydrogenase Kinases (PDKs) and Diseases

Metabolic disorders combined with abnormal PDK activity are often associated with numerous diseases, such as type 2 diabetes, obesity, metabolic disorders, cardiomyopathies, neuropathies, and several types of cancer. PDKs play a key role in metabolic flexibility [65]. They are transcriptionally regulated by insulin, glucocorticoids, thyroid hormone, and fatty acids and play an important role in diabetes and obesity [66]. In type 2 diabetes, the two isoforms PDK2 and PDK4 are induced in a tissue-specific manner. Transcriptional upregulation of PDKs [67–69] decreases the PDH activity in several metabolic disorders, such as diabetes [70–72]. In type 2 diabetes, decreased levels of insulin promote an increase in both PDK4 gene expression and PDK2 mRNA levels. PDK2 and PDK4 mRNAs are upregulated in response to glucose deprivation and fatty acid supplementation. This is reversed by insulin treatment as insulin directly downregulates PDK2 and PDK4 mRNA transcripts [15].

## 6. Interactions between PPAR Gamma and the Canonical Wnt/Beta-Catenin Pathway

### 6.1. PPAR Gamma (Figures 1 and 2)

PPAR alpha, beta/delta, and gamma are ligand-activated transcriptional factors which belong to the nuclear hormone receptor superfamily. PPARs heterodimerize with the retinoid X receptor (RXR). PPAR gamma is expressed in various cell types, such as adipose tissues, muscles, brain, and immune cells. PPAR gamma is involved in the expression of many genes and contributes to glucose homeostasis, insulin sensitivity, lipid metabolism, immune responses, inflammation, and cell fate [73–75]. The net result of the pleiotropic effects of thiazolidinediones (TZDs), a class of PPAR gamma agonists, is improvement of insulin sensitivity [76] in peripheral tissues together with an increase in the glucose-sensing ability of pancreatic beta-cells in diabetic subjects. They improve glucose tolerance and insulin sensitivity in type 2 diabetic patients and in animal models of insulin resistance [77, 78]. Enhanced insulin sensitivity improves peripheral glucose disposal, which decreases the demand for insulin secretion from beta-cells and hepatic glucose production. Effects of TZDs result in increased peripheral glucose use, reduced hepatic glucose output, and, consequently, improvement in overall glycemic control. They act on the promoters of GLUT2 and beta-glucokinase (GK) in pancreatic beta-cells and liver. In adipose tissue, several genes are under the transcriptional control of PPAR gamma, including lipoprotein lipase, acyl-CoA synthetase, fatty acid translocase, and fatty acid transport protein [75]. Dysfunction of PPAR gamma is implied in numerous pathological states such as diabetes, obesity, cancers, and atherosclerosis. TZDs directly activate PPAR gamma and are insulin sensitizing drugs. Some TZDs have been used for treating type 2 diabetes. PPAR gamma also regulates circadian cardiovascular rhythms of blood pressure and heart rate by means of BMAL1 [79, 80]. In cultured muscle cells, PPAR alpha and delta agonists specifically upregulate the expression of PDK4 mRNA, whereas PPAR gamma activation selectively decreases PDK2 mRNA [15]. The PPAR alpha agonist WY-14,643 increases PDK4 mRNA levels in Morris hepatoma 7800 C1 cells [67]. In the diabetic heart, PPAR alpha activity and its downstream targets are upregulated, which leads to a dramatic increase in both fatty acid uptake and oxidation [81] and decreases the mitochondrial pyruvate degradation by upregulating PDK2 and 4.

### 6.2. PPAR Gamma Agonists Induce Beta-Catenin Inhibition in Several Cellular Systems

The functional crosstalk between PPAR gamma and the canonical Wnt/beta-catenin signaling involves the TCF/LEF binding domain of beta-catenin and a catenin binding domain (CBD) within PPAR gamma. In cells that express an APC-containing destruction complex, activation of PPAR gamma induces CPD (Figure 1). TZDs induce a reduction in the cytoplasmic level of beta-catenin in both adipocytes [1] and hepatocytes [3]. PPAR gamma inhibits osteoblastogenesis, promotes adipogenesis, and suppresses the Wnt/beta-catenin pathway during adipogenesis [4, 10]. Conversely Wnt/beta-catenin signaling activation inhibits PPAR gamma and leads to osteogenesis [4].

### 6.3. Inhibition of Canonical Wnt/Beta-Catenin Pathway Induces Activation of PPAR Gamma in Several Cellular Systems

Inhibition of Wnt/beta-catenin signaling and upregulation of PPAR gamma have been reported in ARVC [12, 13]. Gamma-catenin presents structural similarities with beta-catenin [31]. In transgenic mice, gamma-catenin translocates to the nucleus, competes with beta-catenin, and inhibits the canonical Wnt/beta-catenin signaling through the TCF/LEF transcription factors [82, 83]. This results in enhancing adipogenesis, thus summarizing the phenotype of the human ARVC [11–13].

### 6.4. Inactivation of PPAR Gamma and Activation of the Wnt/Beta-Catenin Pathway in Colon Cancer

Beta-catenin-TCF/LEF signaling is activated in colon cancer [84]. Nuclear accumulation of beta-catenin, a marker of poor prognosis, drives cancer cell proliferation. Activation of Wnt signaling can occur via* APC* gene mutations and this enables development of colon cancer [85]. In colon cancer cells, activation of the Wnt/beta-catenin pathway decreases PPAR gamma activity [86]. Beta-catenin can also interact with RXR alpha. In* APC*- and* p53*-mutated colorectal cancer cells, RXR agonists inactivate beta-catenin via RXR alpha. RXR alpha-mediated inactivation of oncogenic beta-catenin occurs in parallel with a reduction in cell proliferation [87]. Mutations in PPAR gamma are linked with human colon cancer [88]. In normal untransformed cells, PPAR gamma induces CPD through both the CBD of PPAR gamma and the TCF binding domain of beta-catenin (Figure 1). In transformed cells, there is no oncogenic beta-catenin degradation. In colon carcinogenesis, PPAR gamma can suppress tumorigenesis by downregulating the oncogene beta-catenin [89]. An early treatment by means of PPAR gamma agonists, and before the onset of carcinogenesis, might prevent tumor development. In many cell types, PPAR gamma agonists induce antitumorigenic effects, probably due to their antiproliferative and prodifferentiation effects. Troglitazone inhibits development of tumors that are derived from colon cancer cells [90]. In transplantable tumors derived from human colon cancer cells, troglitazone induces a significant reduction of growth. Troglitazone fed to rodents decreases the formation of aberrant crypt foci, which is an early stage in the development of colon carcinoma [91]. Activation of PPAR gamma induces CPD in cells that express an APC-containing destruction complex although the oncogenic beta-catenin inhibits the expression of PPAR gamma target genes [6]. Mutations in the TCF/LEF binding domain of an oncogenic beta-catenin leads to both decreased interaction with PPAR gamma and inhibition of PPAR gamma activity [6]. Conversely, in some cases, PPAR gamma activation induces deleterious procarcinogenic effects. Thus, in APC* Min* mice, used as a model for human familial adenomatous polyposis, TZDs increase the number of colon tumors [92, 93]. Numerous studies on cancer and PPAR gamma have focused on the potential for employing PPAR gamma agonists in cancer treatment. As a monotherapy, PPAR gamma agonists have induced little success in clinical trials. Results have been shown promise with combined treatments in culture and animal models. A role for PPAR gamma as a tumor suppressor and inducer of differentiation of cancer stem cells has also been investigated. Various conclusions concerning the prevalence of PPAR gamma mutations in cancer have been observed [94].

### 6.5. Inactivation of PPAR Gamma and Activation of the Wnt/Beta-Catenin Pathway in Type 2 Diabetes

TZDs are potent insulin-sensitizers and certain TZDs represent a therapeutic target for the treatment of type 2 diabetes. However, the involvement of PPAR gamma in numerous pathways generates negative side-effects after PPAR gamma activation by TZDs in tissues or cells not concerned by the disease [95]. PPAR gamma enables activation of GLUT2 and GK in liver and beta-cells and contributes to the beneficial effects induced by TZDs, which improve glucose homeostasis in type 2 diabetic patients. Moreover, dominant-negative mutation in the* PPAR gamma* gene is associated with severe hyperglycemia in patients. This provides a genetic link between PPAR gamma and type 2 diabetes [96]. Humans with dominant-negative mutations in PPAR gamma manifest partial lipodystrophy and severe peripheral and hepatic insulin resistance [97]. Expression of TNF-alpha, which induces insulin resistance, is reduced by PPAR gamma ligands, suggesting that the insulin-sensitizing effect of TZDs is related to its anti-inflammatory properties [98]. PPAR gamma has a significantly lower expression in obese type 2 diabetics than in nondiabetic obese subjects [99].

The Wnt/beta-catenin signaling pathway is involved in diabetes mellitus [100]. Wnt signaling and TCF7L2 are negative regulators of hepatic gluconeogenesis, and TCF7L2 belongs to the downstream effectors of insulin in hepatocytes [101]. Wnt/beta-catenin may represent a link between diabetes and cancer, due to the strong genetic association between specific polymorphisms in the TCF7L2 (TCF4) gene and diabetes [102, 103]. TCF7L2 polymorphisms enhance the risk of developing type 2 diabetes [104–106]. Mutations in LRP5 lead to the development of diabetes and obesity [107]. The Wnt pathway is involved in glucose-induced insulin secretion [108] and production of the incretin hormone glucagon-like peptide-1 [109–111]. Polymorphisms in Wnt5B are associated with a higher risk of developing type 2 diabetes [112]. Otherwise, the human LRP5 gene maps within the IDDM4 region on chromosome 11q13 [113, 114]. Conversely, TCF7L2 knockdown increases human pancreatic beta-cell apoptosis and reduces beta-cell proliferation and glucose-stimulated insulin secretion [115].

## 7. Conclusions

PPAR gamma is downregulated while the canonical Wnt/beta-catenin pathway is upregulated in both type 2 diabetes and colon cancer. Wnt activates some crucial metabolic key enzymes, such as PDKs in the two pathologies. In colon cancer, this leads to aerobic glycolysis or the Warburg effect. Decreased PDH activity by upregulated PDK modifies metabolic flexibility that is, the capacity of the cell to adjust glucose and fatty acid oxidation. Competition between glucose and fatty acids for oxidation occurs at the level of the PDH complex, whose activity is decreased by PDKs. In colon cancer, partial deviation of pyruvate toward lactate contributes to protein synthesis, which are required for cell growth and proliferation. These major metabolic alterations induced by upregulated Wnt/beta-catenin signaling and downregulated PPAR gamma may partly account for the frequently encountered association between type 2 diabetes and colon cancer.

## Abbreviations

APC: Adenomatous polyposis coli
ARVC: Arrhythmogenic right ventricular dysplasia/cardiomyopathy
CPD: Beta-catenin proteasomal degradation
CBD: Catenin binding domain
Dsh: Dishevelled
EMT: Epithelial-mesenchymal transition
Fzd: Frizzled
GK: Glucokinase
GLUT: Glucose transporter
GSK-3beta: Glycogen synthase kinase-3beta
IGF-1: Insulin growth factor
LDH-A: Lactate dehydrogenase-A
LRP5/6: LDL receptor-related protein 5/6 coreceptors
LRP5/6: Low-density lipoprotein receptor-related protein 5/6
MCT-1: Monocarboxylate lactate transporter-1
PPAR gamma: Peroxisome proliferator-activated receptor gamma
PGC-1 alpha: Peroxisome proliferator-activated receptor-gamma coactivator-1 alpha
PDH: Pyruvate dehydrogenase
PDK: Pyruvate dehydrogenase kinase
RXR Alpha: Retinoid X receptor alpha
TCF/LEF: T-cell factor/lymphoid enhancer factor
TZD: Thiazolidinedione
TCA: Tricarboxylic acid.
